# Effect of Short-Term Vitamin D3 Supplementation on Blood Pressure in Patients With Hypertension: A Pilot Study From a Tertiary Care Hospital

**DOI:** 10.7759/cureus.106876

**Published:** 2026-04-12

**Authors:** Santhoshakumari T M J, Mathivanan J, Ramachandran K, Akash M

**Affiliations:** 1 Department of Biochemistry, Mahatma Gandhi Medical College and Research Institute, Sri Balaji Vidyapeeth, Puducherry, IND; 2 Department of Emergency Medicine, Sri Venkateshwaraa Medical College Hospital and Research Centre, Puducherry, IND; 3 Department of Biochemistry, Takshashila Medical College, Takshashila University, Tindivanam, IND; 4 Department of Medicine, Mahatma Gandhi Medical College and Research Institute, Sri Balaji Vidyapeeth, Puducherry, IND

**Keywords:** blood pressure, cardiovascular disease, essential hypertension, intervention, vitamin d3

## Abstract

Background

Essential hypertension is a major risk factor for cardiovascular disease. Emerging evidence suggests that vitamin D deficiency may contribute to the development of essential hypertension through mechanisms involving the renin-angiotensin-aldosterone system and vascular endothelial dysfunction. However, the effect of vitamin D3 supplementation on blood pressure remains controversial. Hence, this study aimed to evaluate the effect of short-term oral vitamin D3 supplementation on blood pressure in patients with essential hypertension.

Methodology

This prospective, interventional pilot study included 30 adult patients diagnosed with essential hypertension. All participants received oral vitamin D3 (250 IU daily) for 12 weeks, in addition to stable antihypertensive therapy. Antihypertensive medications (including angiotensin-converting enzyme inhibitors, angiotensin receptor blockers, calcium channel blockers, and diuretics) were continued without any dose modification during the study period. Systolic blood pressure, diastolic blood pressure, and serum 25-hydroxyvitamin D levels were measured at baseline and after 12 weeks of intervention. Data were expressed as median (interquartile range). The Wilcoxon signed-rank test was used for paired comparisons.

Results

Among the 30 participants, 12 (40%) had normal vitamin D levels, 11 (36.7%) had insufficiency, and seven (23.3%) had deficiency at baseline. After 12 weeks of vitamin D3 supplementation, systolic blood pressure decreased from 133 (130-138) mmHg to 132 (130-136) mmHg, and diastolic blood pressure decreased from 94 (92-96) mmHg to 92 (90-95.5) mmHg (p < 0.001 for both). Serum 25-hydroxyvitamin D levels increased from 27 (20.25-31) ng/mL at baseline to 28 (23-32) ng/mL after supplementation in essential hypertension patients (p < 0.001). Despite being statistically significant, the absolute reductions in blood pressure were small.

Conclusions

Short-term vitamin D3 therapy was associated with a small reduction in blood pressure of uncertain clinical significance among patients with essential hypertension. Due to the absence of a control group, causality cannot be established. These findings should be considered exploratory, and larger randomized controlled studies are necessary to confirm these results.

## Introduction

Essential hypertension is a major public health concern and one of the leading risk factors for cardiovascular disease and mortality worldwide, including in India. It is estimated that about 1.4 billion adults globally and about 188 million adults in India have hypertension. The prevalence continues to rise (estimated at about two-thirds of adults), especially in low- and middle-income countries [1].

Vitamin D has received increasing attention in recent years for its potential role in cardiovascular health. Patients with high blood pressure have been found to have decreased vitamin D levels. Experimental studies have shown that vitamin D may affect blood pressure regulation via various mechanisms, such as by suppressing the renin-angiotensin-aldosterone system, changing the vascular smooth muscle cells’ proliferation, and enhancing endothelial function [2-8].

Observational studies have indicated an inverse association between serum 25-hydroxyvitamin D levels and blood pressure [5,9]. Conversely, clinical investigations assessing the impact of vitamin D3 supplementation on blood pressure have yielded incongruent findings [5,6,9-12]. Some studies have shown a small decrease in blood pressure following vitamin D3 administration [5,6,12-14]. However, others have found no significant effect [9,15-17]. These discrepancies could be attributed to variations in dosage of vitamin D3 supplementation, baseline vitamin D levels of the participants, treatment duration, and the specific characteristics of the study groups. In addition, the dosage of vitamin D3 used across studies varies widely, ranging from low daily doses (400-1,000 IU/day) to higher-dose supplementation (≥2,000 IU/day or intermittent bolus therapy). The present study utilized a relatively low daily dose of 250 IU compared to most previous studies.

Vitamin D deficiency remains highly prevalent in India, despite people getting abundant sunlight exposure. This is likely due to factors such as limited outdoor activity, increased skin color, dietary habit patterns, and their overall lifestyle [18-21]. Given the high prevalence of both hypertension and vitamin D deficiency in India, studying this association in the Indian population is of particular clinical relevance. Therefore, this pilot study was undertaken to assess the effect of short-term oral vitamin D3 supplementation on blood pressure in patients with essential hypertension.

## Materials and methods

Study design

This prospective, single-arm, interventional pilot study was conducted in collaboration between the Department of Medicine and the Department of Biochemistry at Sri Lakshmi Narayana Institute of Medical Sciences, Puducherry, India, from April 2018 to July 2018. This was designed as a single-arm exploratory pilot study without a control group to generate preliminary data and assess feasibility. The Institutional Ethics Committee (Human Studies) approved the study protocol before commencement (approval number: NO.IEC/C-P/01/2018). Written informed consent was obtained from all study participants before enrollment.

Study population

**Figure 1:**
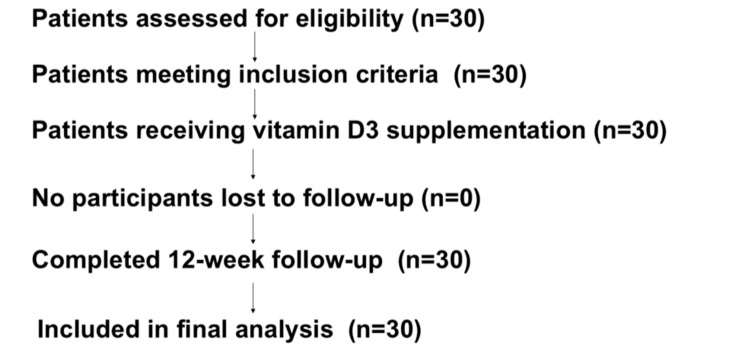
Patient selection flow diagram illustrating the enrolment and follow-up of study participants.

A total of 30 adult patients aged ≥18 years who were diagnosed with essential hypertension were recruited in the study using a consecutive sampling method. The process of patient selection and inclusion is illustrated in Figure 1.

Inclusion and exclusion criteria

Adult patients aged ≥18 years with diagnosed essential hypertension (defined as systolic blood pressure ≥130/90 mmHg and/or diastolic blood pressure ≥90 mmHg on clinical evaluation) who were clinically stable and receiving regular antihypertensive therapy were included.

Patients with secondary hypertension, severe or uncontrolled hypertension, a history of cardiovascular events, chronic liver disease, chronic kidney disease, pregnancy or lactation, endocrine disorders affecting vitamin D metabolism, and those currently receiving vitamin D3 supplementation before enrollment were excluded from the study.

Intervention

All participants were clinically stable and on regular follow-up for essential hypertension. They received oral vitamin D3 supplementation at a dose of 250 IU once daily for 12 weeks. All participants continued their existing antihypertensive medications (including angiotensin-converting enzyme inhibitors, angiotensin receptor blockers, calcium channel blockers, and diuretics) throughout the study period without any change in drug class or dosage. Medication adherence was assessed based on patient self-report during follow-up visits.

During the study period, participants were instructed to maintain their usual diet, physical activity, and daily lifestyle habits. There were no special changes made with respect to salt intake, sunlight exposure, or physical exercise. No participants were lost to follow-up, and all enrolled participants were included in the final analysis.

Measurement of blood pressure

Blood pressure was determined in a clinical setting using a calibrated automated sphygmomanometer. All study participants were placed comfortably in a seated position and given at least five minutes to relax before taking blood pressure measurements. Two readings were taken during each visit, and the average of the readings was used for analysis. Blood pressure readings were collected from the participants pre-intervention (baseline) and post-intervention (after 12 weeks of oral vitamin D3 supplementation) at the same time every day to maintain consistency. The blood pressure measurements were conducted by trained healthcare professionals with a standardized protocol. Observer blinding was not implemented, indicating a limitation.

Sample collection and biochemical analysis

A 3 mL fasting venous blood sample was drawn from the participants and collected in a serum gel separator vacutainer tube with clot activator. The serum was separated by centrifuging at 3,500 rpm for about 15-20 minutes. The separated serum sample of the participants was used for the estimation of the 25-hydroxyvitamin D level by chemiluminescence immunoassay magnetic bead-acridinium ester technology using the iFlash 1200 Immunoassay autoanalyzer. Serum 25-hydroxyvitamin D concentration was measured at baseline and following 12 weeks of oral vitamin D3 supplementation.

Classification of vitamin D status

The study participants were classified according to baseline serum 25-hydroxyvitamin D levels, following established criteria [22]: normal: above 30 ng/mL; insufficient: 20-30 ng/mL; deficient: below 20 ng/mL.

Consideration of sample size

As this was a pilot interventional study, a formal sample size determination was not undertaken. A total of 30 participants were recruited to provide preliminary data and guide the design of future larger studies.

Data analysis

Data analysis was performed using SPSS version 20.0 (IBM Corp., Armonk, NY, USA). The distribution of continuous variables was assessed for normality using the Shapiro-Wilk test. Variables that followed a normal distribution are presented as mean ± standard deviation (SD), whereas non-normally distributed variables are expressed as median and interquartile range (IQR). As the primary outcome variables were not normally distributed, the Wilcoxon signed-rank test was used for paired comparisons between baseline and post-intervention data. A p-value of below 0.05 was considered statistically significant.

## Results

Table 1 shows the baseline characteristics of study participants according to vitamin D status. Among the 30 patients with essential hypertension, 12 (40%) had normal vitamin D levels, 11 (36.7%) had insufficient vitamin D levels, and the remaining seven (23.3%) had vitamin D deficiency. Overall, five males and seven females had normal vitamin D levels, six males and five females had insufficient vitamin D levels, and three males and four females had deficient vitamin D levels. The mean age of the individuals with normal vitamin D and insufficient vitamin D levels was 42.5 ± 6.5 and 39.6 ± 6.9 years, respectively, and that of individuals with deficient vitamin D levels was 38.6 ± 7.5 years.

**Table 1:** Baseline characteristics of study participants according to vitamin D status.

Parameter	Normal vitamin D (>30 ng/mL) (n = 12)	Insufficient vitamin D (20–30 ng/mL) (n = 11)	Deficient vitamin D (<20 ng/mL) (n = 7)
Age (years), mean ± SD	42.5 ± 6.5	39.6 ± 6.9	38.6 ± 7.5
Male, n (%)	5 (41.7)	6 (54.5)	3 (42.9)
Female, n (%)	7 (58.3)	5 (45.5)	4 (57.1)

Table 2 presents the comparison of blood pressure and serum 25-hydroxyvitamin D levels at baseline and after 12 weeks of supplementation in essential hypertension patients using the Wilcoxon signed-rank test. Serum 25-hydroxyvitamin D levels showed a statistically significant increase from 27 (20.25-31) ng/mL at baseline to 28 (23-32) ng/mL after supplementation in essential hypertension patients (p < 0.001). The median systolic blood pressure significantly decreased from 133 (130-138) mmHg at baseline to 132 (130-136) mmHg after 12 weeks of oral vitamin D3 supplementation (p < 0.001). Similarly, median diastolic blood pressure significantly decreased from 94 (92-96) mmHg to 92 (90-95.5) mmHg at baseline to after vitamin D3 oral supplementation in essential hypertension patients (p < 0.001). Despite being statistically significant, the absolute reductions in systolic (1 mmHg) and diastolic (2 mmHg) blood pressure were small.

**Table 2:** Comparison of blood pressure and vitamin D levels at baseline and after 12 weeks of supplementation. Data are presented as median (interquartile range). Statistical analysis was performed using the Wilcoxon signed-rank test. *: Wilcoxon signed-rank test.

Blood pressure	Baseline (mmHg)	After 12 weeks (mmHg)	P-value
Systolic blood pressure (mmHg)	133 (130–138)	132 (130–136)	<0.001*
Diastolic blood pressure (mmHg)	94 (92–96)	92 (90–95.5)	<0.001*
25-hydroxyvitamin D levels (ng/mL)	27 (20.25–31)	28 (23–32)	<0.001*

**Figure 2:**
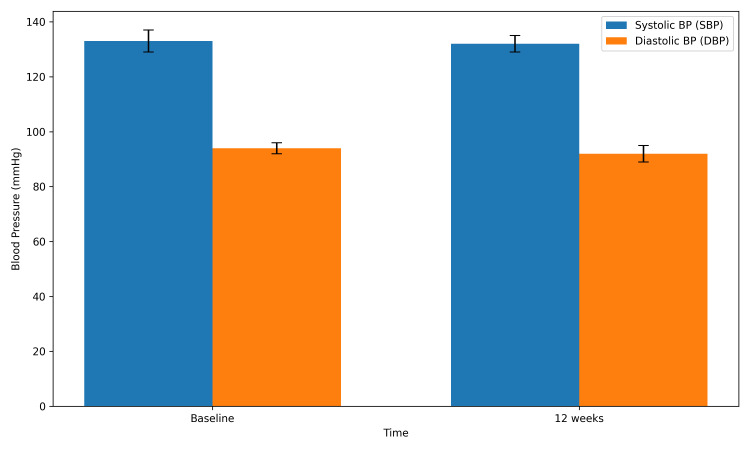
Change in systolic and diastolic blood pressure with interquartile range from baseline to 12 weeks. The bar chart represents the median systolic and diastolic blood pressure (BP) values at baseline and after 12 weeks of vitamin D3 supplementation. Error bars indicate interquartile range (IQR). A small reduction in BP was observed following the intervention.

A graphical representation of changes in systolic and diastolic blood pressure, including IQRs, is shown in Figure 2. Subgroup descriptive analysis suggested a greater trend toward blood pressure reduction among participants with vitamin D insufficiency and deficiency compared with those with normal vitamin D levels. However, no formal subgroup statistical analysis was performed due to the limited sample size.

## Discussion

This prospective pilot study demonstrated that short-term vitamin D3 supplementation was associated with a small reduction in both systolic and diastolic blood pressure in patients with essential hypertension. However, the absolute magnitude of change (1 mmHg systolic and 2 mmHg diastolic) was minimal and of uncertain clinical significance. The relatively low dose of vitamin D3 (250 IU/day) used in this study may partly explain the small magnitude of blood pressure reduction observed.

Our findings are consistent with previous systematic reviews and meta-analyses, demonstrating a small reduction in blood pressure after oral vitamin D treatment, especially in those with pre-existing vitamin D deficiency [5,6,9]. On the contrary, research conducted on vitamin D-sufficient individuals has mostly shown little or negligible clinically significant reduction in blood pressure [12,17].

Vitamin D has been proposed to influence blood pressure regulation through biological pathways, such as by lowering the activity of the renin-angiotensin-aldosterone system and by improving the endothelial function [2-8]. Yet, such mechanisms were not directly investigated in this investigation, which prevented any causal inferences about these pathways.

Even though a statistically significant decrease in blood pressure was observed, the minimal effect size indicates that the therapeutic significance of vitamin D supplementation as an antihypertensive approach is still ambiguous. The observed variations might indicate regression to the mean, inherent variability in blood pressure, or other unmeasured confounding variables.

Subgroup analyses indicated a more significant decrease in blood pressure among patients with vitamin D insufficiency or deficiency. However, owing to the limited sample size and absence of rigorous subgroup analysis, these results must be regarded with careful consideration.

Overall, the outcomes obtained from this study should be considered preliminary. More extensive and well-structured randomized controlled studies are necessary to ascertain whether vitamin D supplementation has a clinically significant impact on blood pressure management.

The present study was intentionally designed as a single-arm, pilot, interventional study without a control group. The primary objective was to generate preliminary data and assess feasibility in a real-world clinical setting rather than to establish causality. Inclusion of a control group was not undertaken due to the exploratory nature of the study and practical constraints. However, the absence of a comparator group remains a significant limitation, as it restricts causal interpretation and does not fully account for potential confounding factors.

Limitations

This study has numerous significant limitations. First, the limited sample size and shorter follow-up period likely affect the generalizability of the results. Second, the absence of a control group hinders causal inference, and the observed changes may be assigned to regression toward the mean, placebo effects, or intrinsic variability in blood pressure. Third, potential confounding variables such as sunlight exposure, dietary salt intake, physical activity, and antihypertensive medications were not meticulously monitored or assessed. Fourth, blood pressure measurements were obtained in a clinical setting without ambulatory monitoring, potentially introducing uncertainty in the findings. The relatively low dosage of vitamin D3 supplementation (250 IU daily) may have limited the reported response amplitude. Finally, the molecular pathways, especially the activity of the renin-angiotensin-aldosterone system and endothelial function, were not investigated. These challenges must be considered while evaluating the results of this pilot study.

## Conclusions

Short-term vitamin D3 supplementation was associated with a small reduction in blood pressure among patients with essential hypertension; however, the clinical significance of this effect remains uncertain. Due to the single-arm design and lack of a control group, causality cannot be determined. These findings should be considered preliminary and contribute to hypothesis formulation. Furthermore, more large-scale randomized controlled trials are necessary to clarify the effect of vitamin D supplementation on blood pressure regulation and ascertain its therapeutic significance.
